# A biallelic loss‐of‐function *PDIA6* variant in a second patient with polycystic kidney disease, infancy‐onset diabetes, and microcephaly

**DOI:** 10.1111/cge.14187

**Published:** 2022-07-18

**Authors:** Elisa De Franco, Matthew N. Wakeling, Russel D. Frew, James Russ‐Silsby, Catherine Peters, Stephen D. Marks, Andrew T. Hattersley, Sarah E. Flanagan

**Affiliations:** ^1^ Institute of Biomedical and Clinical Science, University of Exeter College of Medicine and Health Exeter UK; ^2^ Department of Pediatric Endocrinology Great Ormond Street Hospital for Children London UK; ^3^ Department of Paediatric Nephrology Great Ormond Street Hospital for Children NHS Foundation Trust London UK; ^4^ NIHR Great Ormond Street Hospital Biomedical Research Centre University College London Great Ormond Street Institute of Child Health London UK

**Keywords:** infancy‐onset diabetes, microcephaly, PDIA6, polycystic kidney disease, transcript, whole genome sequencing

## Abstract

We report a second patient with intrauterine growth retardation, congenital polycystic kidney disease, infancy‐onset diabetes, microcephaly, and liver fibrosis caused by a homozygous *PDIA6* loss‐of‐function variant. Our study further defines the genetic and clinical features of this rare syndromic form of infancy‐onset diabetes.

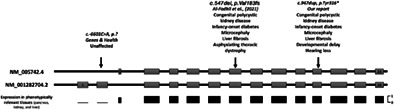

## PEER REVIEW

The peer review history for this article is available at https://publons.com/publon/10.1111/cge.14187.

## ETHICS STATEMENT

Informed consent was obtained from the patient's parents for genetic testing.

To the Editor:

Dysregulation of the endoplasmic reticulum (ER) stress response is an important cause of human disease. Pathogenic variants in at least 10 different ER stress response genes cause syndromic diabetes, with onset ranging from the neonatal period to early adulthood.^1^

Following the publication by Al Fahdli et al^2^ describing a child with asphyxiating thoracic dystrophy and neonatal diabetes caused by a homozygous *PDIA6* variant, NM_001282704:p.Val235fs, we report a second individual with infancy‐onset diabetes and extra‐pancreatic features caused by a pathogenic variant in *PDIA6*.

The male proband was the first‐born child to consanguineous, Middle Eastern parents with a history of multiple pregnancy losses. He was born at 32 weeks gestation weighing 1.4 kg, following antenatal detection of oligohydramnios and echogenic kidneys. He was initially ventilated for 5 days and received two doses of surfactant. Detailed clinical assessment at 3 months noted microcephaly (head circumference ‐6.8SD at 8 months), large polycystic kidneys, hypertension, bilateral inguinal hernias, and umbilical hernia. At 6 months, he was diagnosed with developmental delay, bilateral sensorineural hearing loss, hypotonia, visual impairment, and steatorrhea. A right nephrectomy was performed. An ultrasound scan showed probable fibrotic changes in the liver. Insulin‐dependent diabetes was diagnosed at 8 months. He died in end stage renal failure at 18 months.

**FIGURE 1:**
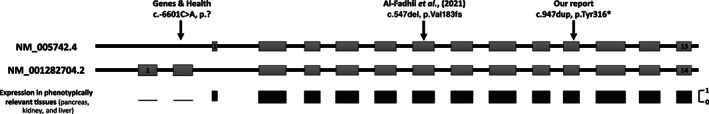
Schematic representation of the *PDIA6* transcripts NM_005742.4 and NM_001282704.2 and their expression across tissues. Mean transcript expression (min = 0, max = 1) of each exon shown in black (data from GnomAD). Variant annotation according to NM_005742.4 transcript shown at the top.

Targeted next‐generation‐sequencing of all known monogenic diabetes genes did not identify a causative variant. Whole‐genome‐sequencing of the proband (samples from parents and previous pregnancies were not available) identified a novel *PDIA6* homozygous stop‐gain variant [NM_001282704:p.Tyr368*, NM_005742:p.Tyr316*] (Figure 1).

Since at the time of the analysis biallelic *PDIA6* variants had not been reported, we investigated the presence of biallelic *PDIA6* loss‐of‐function variants in the GnomAD and Genes and Health (G&H) databases to try and establish causality. There were no homozygous *PDIA6* loss‐of‐function variants in GnomAD, but one homozygous stop‐gain variant [NM_001282704:p.Ser51*] was present in one unaffected individual in G&H, leading us to conclude that the variant was unlikely to be causative. The report by Al Fahdli et al prompted us to reassess this evidence and we found that the variant reported in G&H affects 4 of the 6 *PDIA6* transcripts (including the longest transcript, NM_001282704, Figure 1). The shorter transcript NM_005742, the most abundantly expressed isoform across tissues, was not affected by the G&H variant, but would be degraded because of the variants identified in our patient and in the case reported by Al Fahdli et al. This is consistent with *PDIA6* loss‐of‐function variants affecting the NM_005742 transcript being disease‐causing.

The phenotype of our proband closely resembled the case described by Al Fahdli et al, with intrauterine growth retardation, congenital polycystic kidney disease, infancy‐onset diabetes, microcephaly, and liver fibrosis. Asphyxiating thoracic dystrophy was not detected in our patient.

*PDIA6* variant analysis in 102 diabetic individuals diagnosed <12 months in whom all known genetic aetiologies had been excluded did not identify any additional cases. This suggests that *PDIA6* is an extremely rare genetic subtype of infancy‐onset diabetes. Nonetheless, an early genetic diagnosis of *PDIA6*‐disease is important for medical management and family counselling. We recommend that *PDIA6* is added to gene panels for polycystic kidney disease, infancy‐onset diabetes, and microcephaly.

PDIA6 plays an important role in ER stress regulation through interaction with the kinase PERK^3^ (encoded by *EIF2AK3*, pathogenic variants in *EIF2AK3* are a common cause of syndromic neonatal diabetes). *PDIA6* participates in processing of the misfolded proinsulin protein in vitro, suggesting that it is critical for pancreatic beta‐cell function.^4^ Therefore, beta‐cell destruction through misfolded proinsulin accumulation and dysregulated ER stress is a likely mechanism explaining early‐onset diabetes in the two reported patients with *PDIA6* loss‐of‐function variants.

Our report confirms that biallelic loss‐of‐function variants in *PDIA6* cause a congenital multi‐system syndrome with polycystic kidneys, microcephaly, and infancy‐onset diabetes. This study highlights the importance of using the most biologically relevant transcript when assessing variants in novel aetiological genes.

## CONFLICT OF INTEREST

The authors declare no conflict of interest.

## Data Availability

The data that support the findings of this study are available on request from the corresponding author. The data are not publicly available due to privacy or ethical restrictions.

## References

[1] Shrestha N , De Franco E , Arvan P , Cnop M . Pathological β‐cell endoplasmic reticulum stress in type 2 diabetes: current evidence. Front Endocrinol (Lausanne). 2021;12:650158. 33967960 10.3389/fendo.2021.650158 PMC8101261

[2] Al‐Fadhli FM , Afqi M , Sairafi MH , et al. Biallelic loss of function variant in the unfolded protein response gene PDIA6 is associated with asphyxiating thoracic dystrophy and neonatal‐onset diabetes. Clin Genet. 2021;99(5):694‐703. 33495992 10.1111/cge.13930

[3] Eletto D , Boyle S , Argon Y . PDIA6 regulates insulin secretion by selectively inhibiting the RIDD activity of IRE1. FASEB J. 2016;30(2):653‐665. 26487694 10.1096/fj.15-275883 PMC4714551

[4] Gorasia DG , Dudek NL , Safavi‐Hemami H , et al. A prominent role of PDIA6 in processing of misfolded proinsulin. Biochim Biophys Acta. 2016;1864(6):715‐723. 26947243 10.1016/j.bbapap.2016.03.002

